# Association of grass pollen concentration and physical symptoms as well as impairments in day-to-day life in pollen allergy patients

**DOI:** 10.1038/s41598-025-02462-5

**Published:** 2025-05-28

**Authors:** V. Landesberger, J. Huß, K. Grenzebach, D. Nowak, M. Gröger, E. Oppel, B. Schaub, L. E. French, S. Kutzora, C. Quartucci, C. Herr, S. Heinze

**Affiliations:** 1 Bavarian Health and Food Safety Authority, Munich, Oberschleißheim, Erlangen, Germany; 2 Institute and Clinic for Occupational, Social and Environmental Medicine, University Hospital, LMU Munich, Munich, Germany; 3 Comprehensive Pneumology Center (CPC) Munich, Member of the German Center for Lung Research (DZL), Munich, Germany; 4 Department of Otorhinolaryngology, University Hospital, LMU Munich, Munich, Germany; 5 Department of Dermatology and Allergy, University Hospital, LMU Munich, Munich, Germany; 6 Dr von Hauner Children’s Hospital, Department of Pulmonary and Allergy, LMU University Hospital, LMU Munich, Munich, Germany; 7 LMU, Member of the German Center of Lung Research (DZL), Munich, Germany; 8 German Center for Child and Adolescent Health (DZKJ), Dr von Hauner Children’s Hospital, LMU Munich, Munich, Germany

**Keywords:** Epidemiology, Allergy, Patient education, Health care, Public health

## Abstract

Allergic diseases are a major global public health issue, profoundly impacting the daily lives of millions of people worldwide. The aim of this study is to investigate the association between daily grass pollen concentration and daily physical symptoms as well as impairments in day-to-day life in pollen allergy patients in Bavaria, Germany over a period of three-months. Pollen data of the pollen season 2022 were obtained from the electronic pollen information network of Bavaria. We used an app-based questionnaire and developed an index to measure physical symptoms—regarding eyes and nose as well as impairments in day-to-day life including performance, sleep quality and daily activities. For our analyses we used data from 53 patients. The associations were analysed using linear mixed models (LMM). We found a statistically significant association between the level of grass pollen concentration and both the index physical symptoms (*β* = 0.002; *p* < 0.001) and the index *impairments in day-to-day life* (*β* = 0.00064; *p* < 0.048). It is important that patients are well informed about the pollen count as well as their physical symptoms and daily life impairments so that they can manage their allergies effectively and appropriately.

**Supplementary Information:**

The online version contains supplementary material available at 10.1038/s41598-025-02462-5.

## Introduction

Pollen-related diseases, such as allergic rhinitis (AR), have become an increasing public health concern, profoundly impacting the daily lives of millions worldwide. AR affects up to 18.1% of the world’s population. Over recent decades, the prevalence of AR has steadily increased, with approximately 14.8 percent of adults in Germany experiencing this condition at some point in their lives^1–3^.

AR is clinically characterized as a symptomatic condition of the nose and eyes, triggered by IgE-mediated inflammation upon exposure to allergens^4^. This condition is often initiated by allergic reactions to various environmental allergens, particularly pollen from grasses, trees, and weeds, leading to inflammation of the nasal mucosa and conjunctiva in affected individuals^5^.

The impact of climate change and anthropogenic emissions, including nitrogen oxides, particulate matter, and ozone, is expected to exacerbate this issue, further increasing the number of patients with AR^6,7^. Climate change not only extends the pollen season but also enhances the allergenicity of pollen, thereby elevating the risk of sensitization^6–9^. In Bavaria, an extension of the pollen season and an increase in pollen concentrations throughout the year have already been observed^10^.

The immune response to allergens, including grass pollen, can lead to typical symptoms such as sneezing, nasal congestion, runny nose, and itchy, red, and watery eyes^11^. The burden of disease perceived by patients affects their motivation to seek medical care and adhere to treatment. Measures to reduce the complaints of allergic rhinitis include allergen avoidance, pharmacotherapy, immunotherapy and patient education^5,11^. With regard to pharmacotherapy, the use of intranasal corticosteroids (INCS) is generally recommended, while in cases of severe seasonal AR, a combination of INCS and intranasal antihistamines may be beneficial due to the faster therapeutic effect^11,12^, allergen immunotherapy (AIT) on the other hand can provide long-term relief^5^. In order to genuinely improve the complaints of AR, patients should be aware of their allergy and the associated effects.

Furthermore, experimental studies have demonstrated that pollen exposure can impair cognitive processing^11,13^, sleep quality^14,15^, emotional disorders^16^ and participation in leisure activities and hobbies^5,11^ in pollen allergy patients. These studies have already been performed for various pollen types. While previous studies have extensively investigated the immediate physical symptoms of pollen allergies, there is a notable deficiency in research addressing the long-term effects on work related performance, sleep quality, and daily activities. The aim of this study is to investigate the association between daily measured grass pollen concentrations and both daily physical symptoms and impairments in day-to-day life experienced by pollen allergy patients in Bavaria, Germany, in 2022 using longitudinal data.

## Methods

### Study design and population

In early spring 2022, 168 patients with AR in Bavaria, Germany, were recruited for the study. In this observational pilot study, patients recorded their allergic complaints and treatment measures using a mobile application, called the APOLLO app, for 60 days. All patients gave informed consent to take part in the study.

This study included patients with a physician-diagnosed grass pollen allergy. Patients with concurrent physician-diagnosed allergies to rye, plantain, beech, nettle, oak, or pine were excluded from analysis to minimize confounding allergic complaints from intersecting allergic reactions.

Initially, patients completed a questionnaire on their general health characteristics, which included questions about their general state of health, the presence of physician diagnosed allergies, the presence of asthma and atopic dermatitis, whether AIT has already been carried out and whether the patient is a smoker. It is already known that these variables can have an impact on the severity of AR patients’ complaints^5,12^. These variables were considered in the development of statistical models (LMM_1_ & LMM_2_). Afterwards, patients were then asked to enter their symptoms in eyes and nose every day, as well as their impairments in performance, sleep quality and daily activities. We developed this questionnaire ourselves, drawing on current state of research. Given the need for a questionnaire that could be completed daily within the APOLLO app, we developed our own questionnaire based on current research, as existing tools were too extensive and impractical for this format. Additionally, the patients should indicate whether they have taken medication for their allergic symptoms that day. The detailed study design has been described previously^17^.

The Ethics Committee at the Medical Faculty of Ludwig-Maximilian-University Munich, LMU approved the study including its data protection procedures (project nr.: 20–1149). We are confirming that all experiments were performed in accordance with relevant guidelines and regulations. The study was funded by the Bavarian State Ministry of Health and Care.

### Pollen data

Pollen data were obtained from the Electronic Pollen Information Network of Bavaria (ePIN). This network consists of eight electronic pollen monitors (BAA500, Helmut Hund GmbH) located in Altoetting, Feucht, Garmisch-Partenkirchen, Hof, Marktheidenfeld, Mindelheim, Munich and Viechtach. These monitors provide data on pollen counts in 3-h intervals^18^. Patients chose the pollen monitor nearest to their location each day, with the flexibility to select a different monitor when visiting other areas, If the patients stayed outside Bavaria, no data on their complaints were recorded. For the analysis, the average daily grass pollen concentration from the selected monitor was used individually for each patient.

### Data on allergic complaints

Throughout the study period, patients were asked to record their self-reported allergic complaints on a daily basis. The questions on allergic complaints could be answered for the respective day and for the last two days. The app then provided a summary of these reported allergic complaints, giving patients an overview of their allergic complaints across the entire study period. To ensure consistency, all patients were requested to record their allergic complaints for 60 consecutive days.

Patients entered their physical symptoms into the digital questionnaire, specifically rating their eyes and nose symptoms on a four-point scale ranging from “none” (0) to “severe” (3). A daily *physical symptoms* index *[0–3]* was calculated by adding the values of the symptoms and then dividing them by the number of symptoms (symptoms in nose and eyes; divided by the factor two). A higher index indicates more severe symptoms.

Additionally, patients reported their perceived impairments in day-to-day life, which summarizes impairments in performance, sleep quality and daily activities^5^. The guidelines for allergic rhinitis and its impact on asthma (ARIA) uses these impairments to define moderate/severe AR in comparison to mild AR^5^. Patients rated their impairments on a four-point scale, from "not at all" (0) to “extremely” (3) impaired. From these ratings, a daily overall index of *impairments in day-to-day life [0–3]* was calculated by adding the values of the impairments and then dividing them by the number of impairments (impairments in performance, sleep, activities; divided by the factor three). A higher score indicates more severe impairments. Finally, patients were asked daily whether they had taken any medication for allergic symptoms (yes/no).

### Statistical analysis

All data analyses were performed using SAS 9.4 (SAS Institute, NC). No adjustments were made for missing data. Pearson correlation coefficients were calculated to assess the relationships between reported complaints. Days on which patients were not within the vicinity of the pollen monitoring stations were excluded from the analysis. To avoid data biases in local areas due to meteorological factors when developing statistical models, outliers in pollen concentration were capped at 150 pollen/m^3^^19,20^. The German Weather Service (Deutscher Wetterdienst, DWD) defines a high grass pollen concentration as 30 pollen/m^3^ or more^21^. A cut-off value of 150 pollen/m^3^ ensures that outliers do not distort the pollen data.

To investigate the effects of grass pollen concentration on the two indices, linear mixed models (LMM) for nested data were employed. Lag effects were also calculated for 1,2 and 3 day delays using LMM. All covariates were tested for multicollinearity, and variables with at least moderate correlation (r > 0.3) were excluded from the models. Statistical significance was determined at a p-value of less than 0.05.

## Results

### Pollen concentrations

**Fig. 1:**
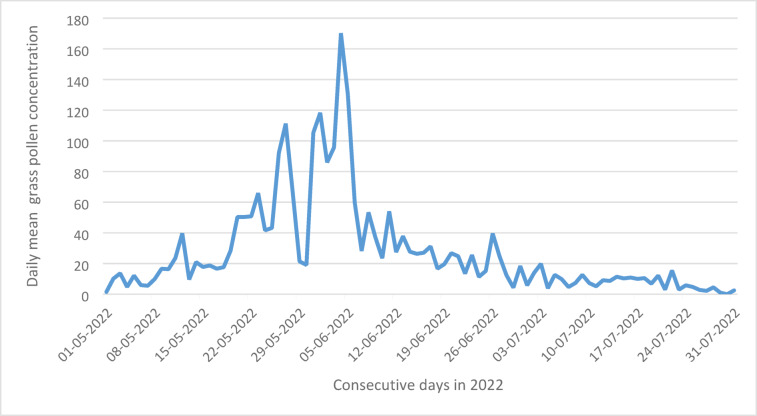
Visualization of daily mean grass pollen concentration (pollen/m^3^) at all measurement sites during the grass pollen season 2022.

Between early May to late July 2022 the average grass pollen concentration was 37.1 pollen per m^3^, with a range of 0 to 852 pollen per m^3^ at all eight measurement sites. Figure 1 shows the course of the daily mean pollen concentrations.

### Demographic characteristics

In total, pollen diary data of 53 patients with grass pollen allergy have been included in our analysis. As shown in Table 1, 68 percent of the patients were women, with an average age of 41.8 years. The general state of health of the patients was predominantly rated as good to very good. 32 percent of the patients received AIT treatment for their allergic complaints in the past. In addition to allergic rhinitis, 45 percent of the patients also had allergic asthma and 43 percent suffered from atopic dermatitis. Overall, 60.4% of participants completed the APOLLO app on more than 80% of the study days.

**Table 1:** Descriptive analysis of study population (N = 53).

Characteristics (N = 53)	Values, n (%)
Gender	
Female	36 (67.9)
Male	17 (32.1)
Age	
Mean Age (Min–Max)	41.8 (18–71)
General health condition	
Very good	14 (26.4)
Good	33 (62.3)
Bad	0 (0.0)
Very Bad	4 (7.5)
Not specified	2 (3.8)
Physician-diagnosed allergic rhinitis	
Intermittend	8 (15.1)
Persistent	43 (81.1)
Severity unspecified	2 (3.8)
Asthma	
Yes	24 (45.3)
No	25 (47.2)
Not specified	4 (7.5)
Atopic dermatitis	
Yes	26 (49.1)
No	19 (35.8)
Not specified	8 (15.1)
Allergen immunotherapy	
Yes	17 (32.1)
No	35 (66.0)
Not specified	1 (1.9)
Smoking status	
Currently smoker	8 (15.1)
Non-smoker	45 (84.9)

### Occurrence of physical symptoms and impairments in day-to-day life

**Fig. 2:**
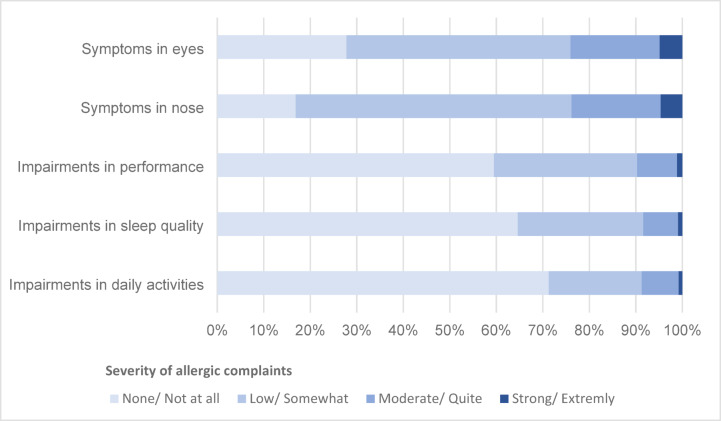
Occurrence of patients’ physical symptoms and impairments in day-to-day life due to their allergy, based on all entries (n = 1439) recorded by allergy patients from May to July 2022.

We analysed 1439 entries in the digital allergy diary. As shown in Fig. 2, nasal symptoms were reported in 83.1% of entries, eye symptoms in 72.2%. Impairments in day-to-day life were mainly found in impairments in performance (40.5%) and sleeping behaviour (35.4%).

On 45.2% of the days when physical symptoms or impairments in day-to-day life were reported, the patients took medication.

We conducted a Pearson correlation test to assess the relationships (Table 2) between physical symptoms and impairments in day-to-day life. All physical symptoms showed a moderate and statistically significant correlation with impairments in everyday life (r = 0.42—0.53). We found a strong correlation between the individual factors of impairments in day-to-day life, in performance, sleep quality and daily activities.

**Table 2:** Pearson correlation coefficients for the relationships between physical symptoms and impairments in day-to-day life.

	Physical symptoms		Impairments in day-to-day life		
	Eyes	Nose	Perfor-mance	Sleep quality	Daily activities
Physical symptoms					
Eyes					
Nose	0.39***				
Impairments in day-to-day life					
Performance	0.45***	0.52***			
Sleep quality	0.42***	0.53***	0.72***		
Daily activities	0.48***	0.49***	0.82***	0.72***	

***p < 0.001.

### Association of grass pollen concentration and the index *physical symptoms* as well as the index *impairments in day-to-day life*

**Fig. 3:**
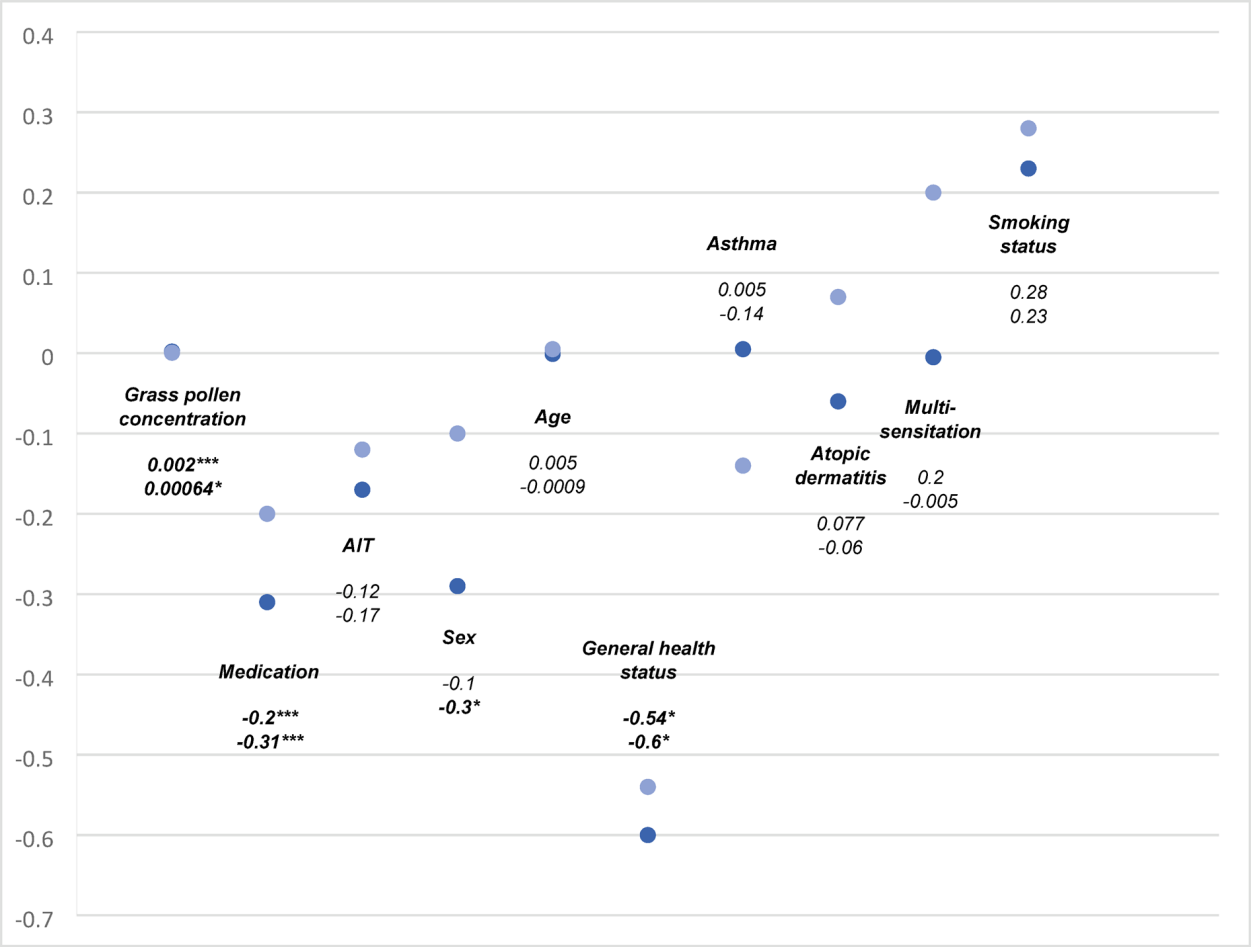
Coefficient Plot of the two main linear mixed models. The scores show the strength of the estimate of the respective parameter of the LMM_1_ for the index *physical symptoms* (dark blue) and the LMM_2_ for the index *impairments in day-to-day life* (light blue). ***p < 0.001. *p < 0.5

We have developed a linear mixed model (LMM_1_) to analyse the nested data (observations = 1439). As seen in Fig. 3, there is a highly significant association of grass pollen concentration and the index *physical symptoms* (*β* = 0.002; *p* < 0.001). For every increase of 10 pollen, the value of the index *physical symptoms* increases by the coefficient 0.02. Taking medication is highly negatively associated with the physical symptoms index. This means taking medication decreases the index *physical symptoms* by 0.31 units. At the beginning of the study, we asked about the general state of health. A good or very good general state of health is negatively associated with the severity of physical symptoms. There is a significant negative association between gender and physical symptoms (*p* = 0.01). This means that men experience fewer physical symptoms compared to women.

We also developed submodels for individual complaints. We found a highly significant association of grass pollen concentration and eye (*β* = 0.0023; *p* < 0.0001) and nasal (*β* = 0.0016; *p* = 0.0009) symptoms.

Furthermore, we also analysed the association between grass pollen concentration and the index impairments in day-to-day life. We have developed a second model (LMM_2_), which shows a significant association of grass pollen concentration on the index *impairments in everyday life* (*β* = 0.00064; *p* < 0.048). The use of medication is highly significantly negatively associated with the severity of the index *impairment in day-to-day life*. Higher medication intake is thus associated with a reduction in the index *impairments in day-to-day life*. In additional submodels, we also looked at the association between grass pollen concentration and impairments in performance, sleep quality and daily activities individually. We found a statistically significant association between grass pollen concentration and performance (*β* = 0.0009; *p* = 0.027), but not with sleep quality (*β* = 0.00077; *p* = 0.052) and daily activities (*β* =  0.00025; *p* = 0.49).

### Lag effects

The association of grass pollen concentration and the index *physical symptoms* was still significant for a lag of 1-day (*β* = 0.0018; *p* < 0.0001) and 2-days ( *β* = 0.001; *p* = 0.0112). No association was found in the LMMs for a 3-day-lag. We did not find any lag effects for the LMM_2_ for impairments in day-to-day life.

## Discussion

### General findings

In this study, we surveyed allergy patients daily about their physical symptoms and impairments in day-to-day life. In general, we found physical symptoms (in eyes and nose) occurred more frequently than impairments in day-to-day life (impairments in performance, sleep quality and daily activities). Physical symptoms and impairments in day-to-day life are correlated; a change in the perceived severity of physical symptoms is also associated with a change in the perceived severity in impairments in day-to-day life, and in reverse. Using a linear mixed model (LMM_1_), we were therefore able to show in our study a highly significant association between the grass pollen concentration and the level of physical symptoms of grass pollen allergic patients, while relevant influencing variables were controlled. This has already been shown in other studies^22–24^. Furthermore, we were able to show a significant association between grass pollen concentration and the severity of impairments in day-to-day life (LMM_2_), while relevant influencing variables were controlled. This has also been proven in experimental studies for impairments in performance^25–27^, impairments in daily activities^28^ and impairments in sleep quality^14^. With our study, for the first time we were able to take a step further by examining and assessing impairments in day-to-day life with the same depth and detail that we applied to the investigation of physical symptoms on a daily basis. The association between grass pollen concentration found was less strong for the index *impairments in day-to-day life* compared to the association of the index *physical symptoms*. The sub-models were not significant when the association between grass pollen concentration and *sleep quality* and the association between grass pollen concentration and *daily activities* were considered. In contrast to experimental studies, in our study allergic complaints were not measured with standardized test procedures, they were self-reported every day. It is possible that it is easier to attribute physical symptoms to pollen allergy in comparison to impairments in day-to-day life, so that self-reported physical complaints are more visible to allergy patients and are mentioned more frequently than impairments in day-to-day life.

We were able to identify a 1-day and 2-day lag effect for the LMM_1_ for the index *physical symptoms*, however none for the LMM_2_ for the index *impairments in day-to-day life*. Other authors have found lag effects for 1–3 days^22,29^. The absence of lag effects for impairments in day-to-day life in our study, may be due to differences in study design and measurement methods. The detectability of lag effects may have been limited by the daily recording of symptoms. Moreover, the participation of patients who were highly motivated to improve their allergy complaints could also have influenced the results.

In this study, we included the intake of medication on the respective day and whether AIT has already been carried out as a possible influencing variable in our model. It is therefore important to consider, that many pollen allergy patients still do not receive adequate medical care. This may be due to the fact that some patients do not take their health condition seriously enough and therefore rarely consult a doctor^30^. Providing appropriate medical treatment can counteract the reported complaints of pollen allergy patients. However, patients should be aware of how AR can affect their health. In addition, an untreated AR can also lead to an extension of an allergic disease. Early onset of AR significantly increases the risk of a shift to the lower respiratory tract, such as asthma, in children and adolescents, supporting the need for early causal therapy^12^. Education of pollen allergy patients and patient self-management has an important impact on the control of AR. Patients who are well-adjusted with their medication experience significantly fewer symptoms compared to those who are not adequately managed^30^.

Future research should explore how technology-assisted self-monitoring can enhance patients’ ability to recognize and attribute their complaints to AR. By visualizing pollen concentrations alongside daily allergic complaints tracking, patients could gain a clearer understanding of the relationship between their allergic complaints and environmental triggers. AR can lead to various ailments in patients^31^. This improved insight may empower patients in their self-management efforts, such as better regulating their medication intake or adopting pollen avoidance strategies, potentially leading to better control of their AR.

### Strengths and limitations

To maintain participants’ motivation over time and ensure a high participation rate, we developed a short, custom questionnaire tailored to the study’s needs. We did not use a standardised questionnaire, even though the assessment of complaints was based on the ARIA guidelines.

In the initial questionnaire, participants were asked whether they had been diagnosed with a pollen allergy by a physician; no study-specific allergy testing was conducted. Data on physical symptoms and impairments in day-to-day life were entirely self-reported. Since we included patients from across Bavaria in the study, incorporating individualized air pollution and weather data into the models was not feasible for such a large area. In future, studies should include these data in their analysis^32^. This study utilized pollen data from a single year, which may limit the generalizability of the findings. It is possible that variations in pollen levels due to an atypical year could affect the association between pollen concentration and the severity of complaints. To minimize the impact of local weather effects on all patients in this study, we examined the dataset for potential outliers. In addition, a control group was not part of the study, we only included pollen allergy patients who were actively interested in being a study participant. This increases the likelihood of selection bias^33^. These individuals may be more engaged with managing their AR and better adjusted to their medication than other patients. Additionally, most of our participants were patients suffering from persistent AR. These individuals may experience more severe symptoms compared to other allergy patients. To increase generalizability for AR patients, future studies should include a larger study population. Due to the limited sample size, which resulted from strict exclusion criteria, the generalizability of the results may be limited. Future studies with larger and more diverse populations are needed to confirm and extend these findings.

The main strength of the study is the Bavaria-wide inclusion of patients, who provided detailed information on their allergic complaints and medication intake on a daily basis over a period of 60 days. The long-term participation of the patients enabled us to utilize longitudinal data for our analyses. The patients were asked to enter their allergic complaints in the digital questionnaire on the same day or two days retrospectively, which reduced recall bias. In addition, real-time pollen measurements are available to us at eight different locations in Bavaria. All patients were able to select a pollen monitor in their individual area, changes in the location of patients within Bavaria were also taken into account when collecting data. This broad coverage of pollen exposure and allergic complaints has provided us with solid data to analyse. The inclusion and exclusion criteria we established ensured that the study population consisted almost exclusively of individuals affected by grass pollen allergy throughout the study. However, due to the exclusion of individuals with multiple overlapping allergies, the results of this study may not be generalizable to this group. Moreover, food allergies or sensitizations to house dust mites or animal dander may have contributed to participants’ reported symptoms.

## Conclusion

AR is a global health burden, which affects patients’ physical symptoms and impairments in day-to-day life. This study provides insights into the association between grass pollen concentration and the daily lives of pollen allergy patients. Our findings confirmed a significant association between grass pollen concentration and both physical symptoms and impairments in day-to-day lives, though the association was stronger for physical symptoms. To enhance clinical practice, it is essential to address the gap in adequate medical care for pollen allergy patients and prioritize patient education on recognizing and managing allergic rhinitis. Advancements in technology-assisted self-monitoring could further empower patients in managing their symptoms and understanding the interplay between environmental triggers and allergic complaints. By building on the findings of this study, we can move closer to improving the quality of life for individuals with AR and advancing the development of targeted interventions.

## Electronic supplementary material

Below is the link to the electronic supplementary material.

Supplementary Material 1

## Data Availability

Pollen data are available on ePIN.bayern.de; the health data analysed during the current study are not publicly available due to the possibility of de-pseudonymization, but are available from the corresponding author on reasonable request.
