# Supplementary material for: Association of grass pollen concentration and physical symptoms as well as impairments in day-to-day life in pollen allergy patients
Source: Sci Rep. 2025 May 28;15:18685. doi: 10.1038/s41598-025-02462-5 (PMC12119830; doi:10.1038/s41598-025-02462-5)
Supplement: Supplementary file 1 — Supplementary Material 1 [file 41598_2025_2462_MOESM1_ESM.docx]

**Supplementary material**: SAS Code

libname library "W:\Daten\..." ;

OPTIONS FMTSEARCH =(library);

**data** Fab1;

set library.analysefb2 ;

**run**;

**data** Fab4;

set Fab1;

PolkonzEFmittel = (Polkonz_1 + Polkonz_2 + Polkonz_3 + Polkonz_4 + Polkonz_5 + Polkonz_6 + Polkonz_7) /**7**;

**run**;

**data** Fab5;

set Fab4;

if c9graeser = **.** then delete;

if c9graeser = **0** then delete;

**run**;

**data** Fab6;

set Fab5;

if c9roggen=**1** then delete;

**run**;

**data** Fab7;

set Fab6;

if c9sonstT = "Wegerich" then delete;

if c9sonstT2 = "Wegerich" then delete;

if c9sonstT = "Spitzwegerich" then delete;

if c9sonstT2 = "Spitzwegerich" then delete;

if c9sonstT = "Buche" then delete;

if c9sonstT2 = "Buche" then delete;

if c9sonstT = "Eiche" then delete;

if c9sonstT2 = "Eiche" then delete;

if c9sonstT = "Kiefer" then delete;

if c9sonstT2 = "Kiefer" then delete;

if c9sonstT = "Brennnessel" then delete;

if c9sonstT2 = "Brennnessel" then delete;

**run**;

**proc** **print** data=Fab7;

var id c9esche c9graeser c9beifuss c9roggen c9ambrosia c9sonstT c9sonstT2 ;

format id;

**run**;

libname library 'W:\Daten\...;

**data** graeser1;

set library.gesamtfinal22_label;

where id in ( **123**, **117**, **156**, **109**, **225**, **240**, **218**, **270**, **198**, **197**, **230**, **112**, **258**, **205**, **250**, **199**, **254**, **175**, **149**, **206**, **304**, **154**, **234**, **276**, **290**, **380**, **317**, **215**, **325**, **330**, **177**, **238**, **259**, **324**, **353**, **214**, **279**, **221**, **251**, **305**, **154**, **283**, **385**, **381**, **122**, **196**, **326**, **342**, **403**, **405**, **285**, **377**, **401**, **402**, **227** );

Hasel=round(Hasel, **1**);

Erle=round(Erle, **1**);

Esche=round(Esche, **1**);

Birke=round(Birke, **1**);

Graeser=round(Graeser, **1**);

Roggen=round(Roggen, **1**);

Beifuss=round(Beifuss, **1**);

Ambrosia=round(Ambrosia, **1**);

**run**;

**proc** **freq** data=graeser1;

tables id;

**run**;

**data** graeser1;

set graeser1;

if not (month(datum) = **4** and year(datum) = **2022**) ;

if not (month(datum) = **3** and year(datum) = **2022**) ;

if not (month(datum) = **8** and year(datum) = **2022**) ;

if not (month(datum) = **9** and year(datum) = **2022**) ;

**run**;

**proc** **print** data=graeser1;

var id datum graeser pl5nase ;

**run**;

**proc** **freq** data=graeser1;

tables id/out=count;

**run**;

**data** pct75;

set count;

if count>**60** then count=**60**;

pct=count/**60**;

format pct percent.;

if pct>=**0.75** then erreicht=**1**;

if pct<**0.75** then erreicht=**0**;

**run**;

**proc** **freq** data=pct75;

tables erreicht;

**run**;

**data** graeser2;

set graeser1;

if pl7leist=**1** then pl7leistUK=**0**;

if pl7leist=**2** then pl7leistUK=**1**;

if pl7leist=**3** then pl7leistUK=**2**;

if pl7leist=**4** then pl7leistUK=**3**;

if pl7leist=**.** then delete;

if pl7schlaf=**1** then pl7schlafUK=**0**;

if pl7schlaf=**2** then pl7schlafUK=**1**;

if pl7schlaf=**3** then pl7schlafUK=**2**;

if pl7schlaf=**4** then pl7schlafUK=**3**;

if pl7schlaf=**.** then delete;

if pl7hobby=**1** then pl7hobbyUK=**0**;

if pl7hobby=**2** then pl7hobbyUK=**1**;

if pl7hobby=**3** then pl7hobbyUK=**2**;

if pl7hobby=**4** then pl7hobbyUK=**3**;

if pl7kont=**.** then delete;

**run**;

**data** graeser3;

set graeser2;

Alltagsbeschwerden= pl7leistUK + pl7schlafUK + pl7hobbyUK;

**run**;

**proc** **freq** data=graeser3;

tables Alltagsbeschwerden;

**run**;

**data** graeser4;

set graeser3;

Qolscoreday= Alltagsbeschwerden/**3**;

**run**;

**proc** **freq** data=graeser4;

tables Qolscoreday;

**run**;

**data** graeser4;

set graeser4;

if c15dauerj=**1** or c15dauerw=**1** then PER=**0**;

if c15dauerj=**2** and c15dauerw=**2** then PER=**1**;

**run**;

**proc** **freq** data=graeser4;

tables PER;

**run**;

**data** graeser4;

set graeser4;

if c38sex = **1** then c38sexUK = **0**;

else if c38sex = **2** then c38sexUK = **1**;

**run**;

**data** graeser4;

set graeser4;

if pl8medi = **1** then pl8mediUK = **0**;

else if pl8medi = **2** then pl8mediUK = **1**;

**run**;

**proc** **freq** data=graeser4;

tables pl8mediUK;

**run**;

**DATA** graeser4;

SET graeser4;

Geburtsdatum = MDY(c40gebmonat, **1**, c40gebjahr);

Alter = INTCK('YEAR', Geburtsdatum, TODAY());

FORMAT Geburtsdatum date9.;

DROP Geburtsdatum;

**RUN**;

**PROC** **MEANS** DATA=graeser4 N MEAN MIN MAX MEDIAN;

VAR ALTER;

**RUN**;

**data** graeser4;

set graeser4;

if c32rauchen <= **2** then c32rauchenUK = **1**;

else c32rauchenUK = **0**;

**run**;

**proc** **freq** data=graeser4;

tables c32rauchenUK;

**run**;

**data** graeser4;

set graeser4;

if pl2ges in (**1**,**2**) then pl2gesUK = **0**;

else if pl2ges in (**3**,**4**) then pl2gesUK = **1**;

**run**;

**proc** **freq** data=graeser4;

tables pl2ges c38sexUK c32rauchenUK/ NOCOL ;

**run**;

**data** graeser4;

set graeser4;

Anzahlpolsens = sum(c9birke, c9hasel, c9erle, c9esche);

Anzahlpolsens = coalesce(Anzahlpolsens, **0**);

**run**;

**data** graeser4;

set graeser4;

if Anzahlpolsens <= **1** then Multisens = **0**;

else Multisens = **1**;

**run**;

**proc** **freq** data=graeser4;

tables Multisens / NOCOL;

**run**;

**data** graeser4;

set graeser4;

if c3asthma = **1** then c3asthmaUK = **1**;

else c3asthmaUK = **0**;

**run**;

**data** graeser4;

set graeser4;

if c3neuro = **1** then c3neuroUK = **1**;

else c3neuroUK = **0**;

**run**;

**PROC** **FREQ** DATA=graeser4;

TABLES c2gesund c3asthmaUK c3neuroUK / NOCOL ;

**RUN**;

**PROC** **FREQ** DATA=graeser4;

TABLES c5probein / NOCOL ;

**RUN**;

**data** graeser4;

set graeser4;

if c17antihis <=**3** then antihisUK=**1**;

else antihisUK=**0**;

**run**;

**PROC** **FREQ** DATA=graeser4;

TABLES antihisUK / NOCOL ;

**RUN**;

**data** graeser4;

set graeser4;

if c11hypo = **1** then c11hypoUK=**1**;

else c11hypoUK=**0**;

**PROC** **FREQ** DATA=graeser4;

TABLES c11hypoUK / NOCOL ;

**RUN**;

**data** graeser5;

set graeser4;

if pl4aug=**1** then pl4augUK=**0**;

if pl4aug=**2** then pl4augUK=**1**;

if pl4aug=**3** then pl4augUK=**2**;

if pl4aug=**4** then pl4augUK=**3**;

if pl4aug=**.** then delete;

**run**;

**data** graeser5;

set graeser5;

if pl5nase=**1** then pl5naseUK=**0**;

if pl5nase=**2** then pl5naseUK=**1**;

if pl5nase=**3** then pl5naseUK=**2**;

if pl5nase=**4** then pl5naseUK=**3**;

if pl5nase=**.** then delete;

**run**;

**data** graeser5;

set graeser5;

physische_Beschwerden= (pl4augUK + pl5naseUK)/**2**;

**run**;

**proc** **freq** data=graeser5;

tables physische_Beschwerden;

**run**;

**proc** **freq** data=graeser5;

tables pl4augUK pl5naseUK;

**run**;

**proc** **freq** data=graeser5;

tables pl7leistUK pl7schlafUK pl7hobbyUK; ;

**run**;

**proc** **freq** data=graeser5;

tables pl8mediUK;

**run**;

**proc** **freq** data=graeser5;

tables pl1pomo;

**run**;

**PROC** **MEANS** DATA=graeser5;

VAR graeser;

OUTPUT OUT=statistiken MEAN=avg MIN=min MAX=max;

**RUN**;

**proc** **corr** data=graeser5 spearman;

var pl4augUK pl5naseUK pl7leistUK pl7schlafUK pl7hobbyUK ;

**run**;

**PROC** **CORR** DATA=graeser5;

VAR pl4augUK pl5naseUK pl7leistUK pl7schlafUK pl7hobbyUK;

WITH pl4augUK pl5naseUK pl7leistUK pl7schlafUK pl7hobbyUK;

**RUN**;

**proc** **print** data=graeser5;

var id datum graeser physische_Beschwerden Qolscoreday ;

**run**;

**data** graeser5;

set graeser5;

if graeser>**150** then graeser=**150**;

**run**;

**data** graeser5;

set graeser5;

if c2gesund <=**2** then c2gesundUK=**1**;

else c2gesundUK=**0**;

**run**;

**proc** **freq** data=graeser5;

tables c2gesundUK ;

**run**;

**PROC** **MIXED** DATA = graeser5;

class ID;

MODEL physische_Beschwerden = graeser pl8mediUK c32rauchenUK multisens c2gesundUK c3neuroUK alter c38sexUK c3asthmaUK c11hypoUK / SOLUTION;

RANDOM INTERCEPT / SUBJECT = ID;

ods output SolutionF=MixedModelPhysisch;

**RUN**;

**data** MixedModelPhysisch;

set MixedModelPhysisch;

Lower_CI = Estimate - **1.96** * StdErr;

Upper_CI = Estimate + **1.96** * StdErr;

**run**;

**proc** **sgplot** data=MixedModelPhysisch;

scatter x=Effect y=Estimate / markerattrs=(symbol=circlefilled);

highlow y=Estimate low=Lower_CI high=Upper_CI / lineattrs=(thickness=**2**);

xaxis label="Effekt";

yaxis label="Koeffizienten";

refline **0** / axis=y lineattrs=(pattern=shortdash);

keylegend / location=inside position=topright across=**1**;

**run**;

**proc** **corr** data=graeser5 spearman;

var pl8mediUK c32rauchenUK multisens c2gesundUK c3neuroUK alter c38sexUK c3asthmaUK c11hypoUK ;

**run**;

**PROC** **MIXED** DATA = graeser5;

class ID;

MODEL pl5naseUK = graeser pl8mediUK c32rauchenUK c2gesundUK multisens c3neuroUK alter c38sexUK c3asthmaUK c11hypoUK/ SOLUTION;

RANDOM INTERCEPT / SUBJECT = ID;

**RUN**;

**PROC** **MIXED** DATA = graeser5;

class ID;

MODEL pl4augUK = graeser pl8mediUK c32rauchenUK c2gesundUK multisens c3neuroUK alter c38sexUK c3asthmaUK c11hypoUK/ SOLUTION;

RANDOM INTERCEPT / SUBJECT = ID;

**RUN**;

**data** graeser5;

set graeser5;

if graeser>**150** then graeser=**150**;

**run**;

**proc** **freq** data=graeser5;

tables Qolscoreday;

**run**;

**PROC** **MIXED** DATA = graeser5;

class ID;

MODEL Qolscoreday = graeser pl8mediUK c32rauchenUK multisens c2gesundUK c3neuroUK alter c38sexUK c3asthmaUK c11hypoUK / SOLUTION;

RANDOM INTERCEPT / SUBJECT = ID;

**RUN**;

**PROC** **MIXED** DATA = graeser5;

class ID;

MODEL pl7schlafUK = graeser pl8mediUK c32rauchenUK multisens c2gesundUK c3neuroUK alter c38sexUK c3asthmaUK c11hypoUK / SOLUTION;

RANDOM INTERCEPT / SUBJECT = ID;

**RUN**;

**PROC** **MIXED** DATA = graeser5;

class ID;

MODEL pl7leistUK = graeser pl8mediUK c32rauchenUK multisens c2gesundUK c3neuroUK alter c38sexUK c3asthmaUK c11hypoUK / SOLUTION;

RANDOM INTERCEPT / SUBJECT = ID;

**RUN**;

**PROC** **MIXED** DATA = graeser5;

class ID;

MODEL pl7hobbyUK = graeser pl8mediUK c32rauchenUK multisens c2gesundUK c3neuroUK alter c38sexUK c3asthmaUK c11hypoUK / SOLUTION;

RANDOM INTERCEPT / SUBJECT = ID;

**RUN**;

**proc** **corr** data=graeser5 spearman;

var pl7leistUK pl7schlafUK pl4augUK pl5naseUK pl6lungUK ;

**run**;

**proc** **corr** data=graeser5 spearman;

var pl7leistUK pl7schlafUK pl7hobbyUK;

**run**;

**data** graeser11;

set graeser5;

lag3_Graeser = lag3(Graeser);

**run**;

**proc** **mixed** data=graeser11;

class ID ;

model physische_Beschwerden = lag3_Graeser pl8mediUK c32rauchenUK multisens c2gesundUK c3neuroUK alter c38sexUK c3asthmaUK c11hypoUK / solution;

random intercept / subject=ID type=un;

**run**;

**data** graeser12;

set graeser5;

lag2_Graeser = lag2(Graeser);

**run**;

**proc** **mixed** data=graeser12;

class ID ;

model physische_Beschwerden = lag2_Graeser pl8mediUK c32rauchenUK multisens c2gesundUK c3neuroUK alter c38sexUK c3asthmaUK c11hypoUK / solution;

random intercept / subject=ID type=un;

**run**;

**data** graeser13;

set graeser5;

lag1_Graeser = lag1(Graeser);

**run**;

**proc** **mixed** data=graeser13;

class ID ;

model physische_Beschwerden = lag1_Graeser pl8mediUK c32rauchenUK multisens c2gesundUK c3neuroUK alter c38sexUK c3asthmaUK c11hypoUK / solution;

random intercept / subject=ID type=un;

**run**;

**proc** **mixed** data=graeser13;

class ID ;

model Qolscoreday = lag1_Graeser pl8mediUK c32rauchenUK multisens c2gesundUK c3neuroUK alter c38sexUK c3asthmaUK c11hypoUK / solution;

random intercept / subject=ID type=un;

**run**;

**proc** **mixed** data=graeser12;

class ID ;

model Qolscoreday = lag2_Graeser pl8mediUK c32rauchenUK multisens c2gesundUK c3neuroUK alter c38sexUK c3asthmaUK c11hypoUK / solution;

random intercept / subject=ID type=un;

**run**;

**proc** **mixed** data=graeser11;

class ID ;

model Qolscoreday = lag3_Graeser pl8mediUK c32rauchenUK multisens c2gesundUK c3neuroUK alter c38sexUK c3asthmaUK c11hypoUK / solution;

random intercept / subject=ID type=un;

**run**;

libname library 'W:\Daten\...;

**data** fb1;

set library.fbfinal22_label;

where id in ( **109**, **117**, **122**, **123**, **149**, **154**, **156**, **175**, **177**, **196**, **197**, **198**, **199**, **205**, **206**, **214**, **215**, **218**, **221**, **225**, **227**, **230**, **234**, **238**, **240**, **250**, **251**,**254**, **258**, **259**, **270**, **276**, **279**, **283**, **285**, **290**, **304**, **305**, **317**, **324**, **325**, **326**, **330**, **342**, **353**, **377**, **380**, **381**, **385**, **401**, **402**, **403**, **405**);

**run**;

**proc** **sort** data=fb1 nodupkey;

by id;

**run**;

**proc** **freq** data=fb1;

tables c38sex;

**run**;

**DATA** fb1;

SET fb1;

Geburtsdatum = MDY(c40gebmonat, **1**, c40gebjahr);

Alter = INTCK('YEAR', Geburtsdatum, TODAY());

FORMAT Geburtsdatum date9.;

DROP Geburtsdatum;

**RUN**;

**PROC** **MEANS** DATA=fb1 N MEAN MIN MAX MEDIAN;

VAR ALTER;

**RUN**;

**data** fb1;

set fb1;

if c38sex = **1** then c38sexUK = **0**;

else if c38sex = **2** then c38sexUK = **1**;

**run**;

**proc** **freq** data=fb1;

tables c38sexUK;

**run**;

**data** fb1;

set fb1;

if c32rauchen= **1** then c32rauchenUK = **1**;

if c32rauchen= **2** then c32rauchenUK = **1**;

if c32rauchen= **3** then c32rauchenUK = **1**;

if c32rauchen= **4** then c32rauchenUK = **0**;

if c32rauchen= **5** then c32rauchenUK = **0**;

if c32rauchen= **6** then c32rauchenUK = **0**;

**run**;

**proc** **freq** data=fb1;

tables c32rauchen c32rauchenUK c11hypo / NOCOL ;

**run**;

**PROC** **FREQ** DATA=fb1;

tables c2gesund;

**run**;

**PROC** **FREQ** DATA=fb1;

tables c46schab;

**run**;

**data** fb1;

set fb1;

if c3asthma = **1** then c3asthmaUK = **1**;

else c3asthmaUK = **0**;

**run**;

**PROC** **FREQ** DATA=fb1;

TABLES c3asthma c3neuro ;

**RUN**;

**proc** **freq** data=fb1;

tables c15dauerw c15dauerj;

**run**;

**data** fb1;

set fb1;

if c15dauerj=**1** or c15dauerw=**1** then PER=**0**;

if c15dauerj=**2** and c15dauerw=**2** then PER=**1**;

**run**;

**proc** **freq** data=fb1;

tables PER;

**run**;
